# Anorexia nervosa admissions during the COVID-19 pandemic: results from a Department of Psychiatry in northern Portugal

**DOI:** 10.1192/j.eurpsy.2022.268

**Published:** 2022-09-01

**Authors:** A. Vieira, P. Nunes

**Affiliations:** Centro Hospitalar Universitário de São João, Psychiatry, Porto, Portugal

**Keywords:** Covid-19, Inpatient care, Anorexia nervosa

## Abstract

**Introduction:**

Emerging evidence suggests that the COVID-19 pandemic had a negative impact on mental health. In particular, patients with Anorexia nervosa (AN) may have faced increased symptom severity.

**Objectives:**

To compare the clinical characteristics of inpatients with AN admitted amidst the COVID-19 pandemic versus the two previous years.

**Methods:**

Retrospective observational study of inpatients admitted between January 2018 and December 2020 in a psychiatry inpatient unit of a tertiary hospital.

**Results:**

There were 11 admissions of patients with AN in 2020 (8 from March onwards), a 22% increase relative to 2019, which in turn saw a 28% increase in admissions relative to 2018. Most patients had an AN diagnosis previous to the pandemic. The majority were undergoing outpatient treatment for over a year. Two patients were admitted within a month of outpatient treatment. There was an increase in admissions through the emergency service in 2020. The most frequent diagnostic was AN binge-eating/purging type in 2020 and 2019, whereas in 2018 the AN restrictive type was dominant. Mean BMI at admission and average length of stay were similar across the three years. Readmission in a 12-month period was 54,5% in 2020 (22,2% in 2019 and 42,9% in 2018).

**Conclusions:**

Despite the widespread impression of a negative impact of the pandemic on AN patients, in our study the clinical characteristics of AN patients admitted in 2020 were mostly similar to the two previous years. Readmissions were higher in 2020, therefore future analysis of data from 2021 might be more enlightening.

**Disclosure:**

No significant relationships.

